# *Escherichia coli* σ^70^ promoters allow expression rate control at the cellular level in genome-integrated expression systems

**DOI:** 10.1186/s12934-020-01311-6

**Published:** 2020-03-05

**Authors:** Artur Schuller, Monika Cserjan-Puschmann, Christopher Tauer, Johanna Jarmer, Martin Wagenknecht, Daniela Reinisch, Reingard Grabherr, Gerald Striedner

**Affiliations:** 1 Christian Doppler Laboratory for Production of Next-level Biopharmaceuticals in E. coli, Department of Biotechnology, University of Natural Resources and Life Sciences, Muthgasse 18, 1190 Vienna, Austria; 2 Boehringer Ingelheim RCV GmbH & Co KG, Dr.-Boehringer-Gasse 5-11, 1120 Vienna, Austria

**Keywords:** Recombinant protein expression, *Escherichia coli*, LacI autoregulation, Tunable expression, σ^70^ promoters, Genome-integrated expression systems

## Abstract

**Background:**

The genome-integrated T7 expression system offers significant advantages, in terms of productivity and product quality, even when expressing the gene of interest (GOI) from a single copy. Compared to plasmid-based expression systems, this system does not incur a plasmid-mediated metabolic load, and it does not vary the dosage of the GOI during the production process. However, long-term production with T7 expression system leads to a rapidly growing non-producing population, because the T7 RNA polymerase (RNAP) is prone to mutations. The present study aimed to investigate whether two σ^70^ promoters, which were recognized by the *Escherichia coli* host RNAP, might be suitable in genome-integrated expression systems. We applied a promoter engineering strategy that allowed control of expressing the model protein, GFP, by introducing *lac* operators (*lacO*) into the constitutive T5 and A1 promoter sequences.

**Results:**

We showed that, in genome-integrated *E. coli* expression systems that used σ^70^ promoters, the number of *lacO* sites must be well balanced. Promoters containing three and two *lacO* sites exhibited low basal expression, but resulted in a complete stop in recombinant protein production in partially induced cultures. In contrast, expression systems regulated by a single *lacO* site and the *lac* repressor element, *lacI*^*Q*^, on the same chromosome caused very low basal expression, were highly efficient in recombinant protein production, and enables fine-tuning of gene expression levels on a cellular level.

**Conclusions:**

Based on our results, we hypothesized that this phenomenon was associated with the autoregulation of the *lac* repressor protein, LacI. We reasoned that the affinity of LacI for the *lacO* sites of the GOI must be lower than the affinity of LacI to the *lacO* sites of the endogenous *lac* operon; otherwise, LacI autoregulation could not take place, and the lack of LacI autoregulation would lead to a disturbance in *lac* repressor-mediated regulation of transcription. By exploiting the mechanism of LacI autoregulation, we created a novel *E. coli* expression system for use in recombinant protein production, synthetic biology, and metabolic engineering applications.

## Background

In industrial recombinant protein production processes, regulation of the gene of interest (GOI) is an important prerequisite. Transcription rates are controlled by the interaction between a promoter and the RNA polymerase (RNAP). This interaction must be understood and externally regulated to provide process control, and thereby, the optimization of product yield and quality. In particular, challenging proteins of interest, like antibody fragments, membrane proteins, or toxic proteins, require low basal expression in non-induced states and a reduced transcriptional activity after recombinant protein induction [1–3]. The final yield of challenging proteins is not only directly determined by the strength of the promoter system but also by further processing steps, such as translation, folding, translocation into the periplasm, and proper disulfide bond formation. The most prominent and well-studied genetic regulatory mechanism is the *lac* operon of *Escherichia coli* [4]. In wild-type *E. coli*, the *lac* repressor protein (LacI) evolved to sense the presence of lactose. In the absence of lactose, LacI forms a homo-tetramer that binds to the *lac* operator site (*lacO*) and represses the transcription of the *lacZYA* operon [5]. Conversely, when lactose or isopropyl β-D-1-thiogalactopyranoside (IPTG, a non-metabolizable structural mimic of allolactose) binds to LacI, it induces a conformational change in the protein structure, and LacI can no longer bind to *lacO* site. This leaves the *lacO* site open to RNAP binding, and thus, transcription can start. The *lacO* sites are DNA sequences with an inverted repeat symmetry [6]. The higher the symmetry, the greater the LacI binding affinity of the operator sequence. An artificial, perfectly symmetric *lacO* (sym-*lacO*) was found to bind LacI with the greatest affinity [7]. In contrast, three wild-type operators *lacO1*, *lacO2*, and *lacO3*, which exhibited approximate symmetry, showed lower affinities, in the following descending order: sym-*lacO* > *lacO1* > *lacO2* > *lacO3* [8]. LacI binds simultaneously to both the primary operator, *lacO1*, and to either *lacO2* or *lacO3* through a DNA-looping mechanism [9]. *LacO2* is located 401 bp downstream of *lacO1*, and *lacO3* lies 92 bp upstream of *lacO1* [10]. Due to their close proximity, the DNA-looping mainly occurs between *lacO1* and *lacO3*, and thus, these sites provide the main gene repression [8]. Consequently, the role of *lacO2* remains unclear. Furthermore, when LacI binds *lacO1* and *lacO3*, it inhibits its own production, because the 3′ end of the *lacI* gene overlaps with *lacO3*. In the repressed state, *lacI* transcription results in a truncated mRNA, which is rapidly degraded by the cell. Due to this autoregulation, the abundance of the LacI tetramer is ~ 40 molecules per cell in induced cells and ~ 15 molecules per cell in non-induced cells [11].

One application of the *lac* regulatory mechanism is known as the pET system, which is currently the most widely used *E. coli* expression system for recombinant protein production [12, 13]. The pET system is based on the specific interaction between the phage-derived, T7-specific RNAP and the strong T7 promoter for the GOI. The recombinase functions of bacteriophage lambda were used for site-directed insertion of the T7 RNAP gene into the *E. coli* chromosome. Expression of the T7 RNAP is controlled by the lacUV5 promoter, a variant of the *lac* promoter that is insensitive to catabolic repression [14]. The addition of IPTG induces the expression of the T7 RNAP at high levels, which in turn, transcribes the target gene under the control of the T7 promoter [13]. This orthogonal expression system offers very high product titers for recombinant proteins that, consequently, can be efficiently produced in *E. coli*. However, the extraordinary strength of the T7 expression system, particularly when combined with high-copy-number plasmids, exerts an extreme metabolic load on the host cells. When the GOI codes for a challenging protein, the stress and metabolic burden often lead to reduced yield, shortened production periods, and even cell death [15, 16].

Plasmid-mediated stress, due to high gene dosages and the expression of antibiotic resistance genes, can be overcome by integrating the GOI into the host chromosome [17, 18]. The high efficiency of the T7 RNAP compensates for low gene dosages and provides high rates of recombinant gene expression [15]. Nevertheless, the high expression rates also cause stress to the cell, which results in reduced growth rates. In a previous study [18] we showed that during continuous production, the genome-integrated T7 expression system became instable approximately 70 h past induction. The reason for this could be found in a mutated T7 RNAP, which led to a faster growing non-producing population [unpublished data]. These non-producing cells grew more rapidly and prevailed over the producing population; this resulted in a massive loss in product yield.

We expected that expression systems that are coupled to the host metabolism would have increased genetic stability, because transcription relies on constitutive phage-derived promoters that are recognized by the σ^70^*E. coli* RNAP, rather than relying on transcription machinery that is orthogonal to *E. coli*. The pQE vectors from Qiagen (Hilden, Germany) provide two *lacO* sites that control the T5_N25_ promoter. The pJexpress 401-406 (T5) vectors from ATUM (Newark, NJ, USA) contain two wild-type *lacO* sites and one symmetric *lacO* site to avoid basal expression. The *E. coli* pAVEway™ expression system from Fujifilm Diosynth Biotechnologies (Hillerød, Denmark) employs two symmetrical *lacO* sites to control the expression of the T7_A3_ promoter. However, all these expression systems are plasmid-based, and thus, they are subject to the obstacles mentioned above, like high gene copy number, plasmid replication, and process instability caused by plasmid loss.

The present study aimed to generate inducible promoters that were recognized by the σ^70^*E. coli* RNAP and were originally derived from two constitutive phage promoters, T5 (T5_N25_) [19–21] and A1 (T7_A1_) [22]. We aimed to investigate their potential transcription efficiency, basal expression rate, and transcription rate control, in genome-integrated expression systems. For transcription rate control, we introduced one [21], two [23], or three *lacO* sites [7], into the promoter sequences. We integrated these into *E*. *coli* strains with wild-type *lacI* and *lacI*^*Q*^ promoters. The *lacI*^*Q*^ promoter is a variant with a single C → T change within the − 35 promoter motif. This mutation causes a tenfold increase in LacI expression [24]. The resulting promoter/operator combinations were investigated to determine expression strength, tunability, and basal expression of the cytosolic model protein, GFPmut3.1 [25]. We also evaluated cell growth in plasmid-based and genome-integrated *E. coli* BL21 expression systems. We reasoned that the addition of *lac* operators on the chromosome in the genome-integrated expression systems might influence the endogenous *lac* operon activity. Therefore, we also measured the LacI levels in selected strains. The production clones were compared in micro-titer fermentations, under fed-batch-like conditions [26], over a production period of 12 h, and they were benchmarked with the T7 RNAP-dependent T7 promoter expression systems.

## Results and discussion

**Fig. 1:**
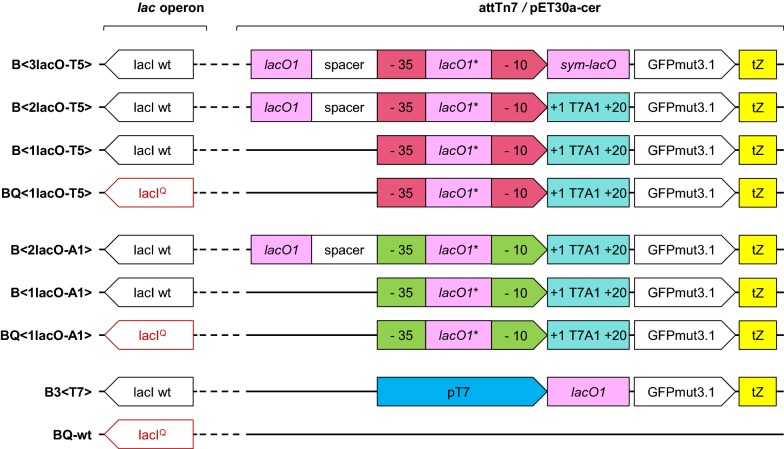
Schematic of GFPmut3.1 expression cartridges controlled by seven different promoter/operator combinations. The cartridges were integrated into the attTN7 site (indicated with*<*pointed brackets*>*) of the *E. coli* BL21 chromosome, or they were cloned into the pET30a-cer vector (indicated with round brackets (), but not shown in this figure). In two promoter/operator combinations, the wild-type *lacI* promoter (B, black) was exchanged with the *lacI*^*Q*^ promoter (BQ, red). LacO1* is a 2-bp truncated version of wild-type *lacO1*. Sym-*lacO* is the perfectly symmetric *lacO*. The native, initially transcribed sequence of the A1 promoter, is labeled +1 T7A1 +20. Transcription is terminated by tZENIT (tZ) [27]. The BL21(DE3) T7 expression system (B3<T7>) is used as a reference. The BQ-wt carried the wild-type sequence, with the *lacI*^*Q*^ promoter

In this study, we investigated the protein expression potential of two modified phage-derived promoters, T5 and A1, which were recognized by the σ^70^*E. coli* RNAP. The promoter sequences were modified to contain one, two, or three *lacO* sites. We created seven promoter/operator constructs combined with the open reading frame of the model protein, GFPmut3.1 (Fig. 1).

### Productivity of σ^70^ dependent promoter/operator combinations

The T7 expression system is known to provide high expression rates, even from a single target gene copy, when integrated into the *E. coli* chromosome. First, we wanted to check whether the same productivity could be reached with σ^70^*E. coli* RNAP-dependent promoters in the same experimental set-up. Therefore, we compared the genome-integrated (indicated with pointed brackets: <>) and plasmid-based (indicated with round brackets) T5 and A1 promoter/operator combination expression systems to the T7 expression system. The cells were grown in fed-batch-like conditions, in micro-titer fermentations, over a period of 22 h. Expression of GFPmut3.1 was induced with 0.5 mM IPTG after 10 h.

In all promoter/operator combinations, the cells maintained growth in the micro-titer fermentations. The average growth rate was µ = 0.05/h, during the 12-h production period. We directly compared average growth rates between the T7 and the σ^70^ promoters (Additional file 1: Figure S1, Additional file 2: Figure S2).

**Fig. 2:**
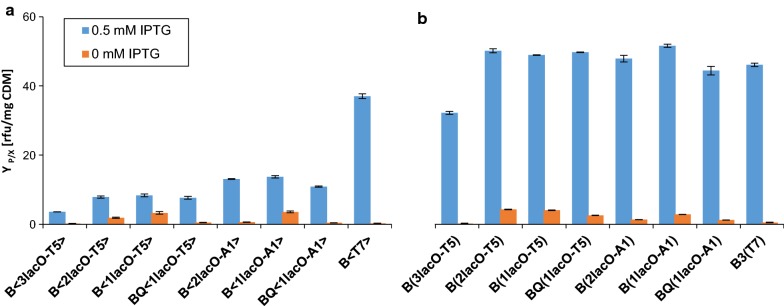
Promoter activities of different promoter/operator combinations, under non-induced (0 mM IPTG) and induced (0.5 mM IPTG) conditions. The specific fluorescence of the reporter protein, GFPmut3.1 (Y_P/X_), is given in relative fluorescence units per mg of cell dry matter [rfu/mg CDM]. This value was used to characterize **a** genome-integrated expression systems and **b** plasmid-based expression systems. Error bars indicate standard error of the mean (n = 3). Expression system names are defined in Fig. 1

On-line fluorescence measurements of the plasmid-based expression systems (Fig. 2b) showed that all promoter/operator combinations, except B(3lacO-T5), expressed comparable amounts of GFPmut3.1. In contrast, with the genome-integrated expression systems (Fig. 2a), we observed quite distinct differences between the different promoter/operator combinations. The A1 expression systems produced 1.5-fold GFPmut3.1 yields compared to the T5 expression systems. These results were consistent with previously published data [20, 21, 28]. In the genome-integrated T7 expression system, induction of GFPmut3.1 expression led to 145 rfu and a specific soluble GFPmut3.1 concentration of ~ 135 mg/g cell dry matter (CDM). The same experiment with the A1 expression systems yielded almost 50 rfu and a GFPmut3.1 concentration of 37 mg/g CDM. A comparison of protein solubility in the plasmid-based and genome-integrated systems indicated that a large proportion of insoluble GFPmut3.1 was produced in the plasmid-based expression systems. Conversely, over 90% of the recombinant protein was soluble in the genome-integrated expression systems (Additional file 3: Figure S3).

The reduced productivity observed with the plasmid-based B(3lacO-T5) and the genome-integrated B<3lacO-T5> might have been due to the presence of the perfectly symmetric *lac* operator (sym-*lacO*) [7], which replaced the initially transcribed sequence (ITS). This symmetric *lacO* could influence promoter escape, and therefore, productivity [29]. This effect was less evident with the plasmid-based 3(lacO-T5) expression system, where the high plasmid copy number compensated for the reduced promoter activity. However, in the genome-integrated expression system, the promoter activity was quite low; therefore, we discarded the 3*lacO* version with the A1 promoter. For the one and two *lacO* promoter/operator combinations, we replaced sym-*lacO* with the native ITS of the A1 promoter (+1 *T7A1* +20). This resulted in a 1.4-fold increase in productivity, in the case of the T5 promoter.

### Basal expression in σ^70^ dependent expression systems

For challenging proteins, even low basal expression can have adverse effects on the host metabolism, or it may even be toxic to the host cell. Hence, in those cases, equipping the host with an expression construct, either plasmid-based or genome-integrated, can be rather difficult. This difficulty is typically represented by the low frequency of transformants or integrants, respectively. Thus, the tightness of gene regulation is an important quality criterion for expression systems.

In the plasmid-based systems, promoters that were controlled by one *lac* operator (1*lacO*) showed the highest basal expression, at a level of ~ 4 rfu/mg CDM, particularly under carbon-limited conditions (Fig. 2b). The addition of a second *lacO* (2*lacO*) or an increase in LacI production, by introducing the *lacI*^*Q*^ promoter, reduced the basal expression of the A1 promoter to 1 rfu/mg CDM. In constructs with the T5 promoter, only the inclusion of three *lac* operators (3*lacO*) reduced the basal expression to almost 0 rfu/mg CDM. In contrast to the plasmid-based expression systems, all genome-integrated systems showed that the promoter/operator combination significantly impacted the system leakiness (Fig. 2a). Both an increase in the number of LacI molecules and the addition of a second *lacO* site reduced the basal expression of A1 expression systems from 4 rfu/mg CDM to nearly no significant background expression. Importantly, productivity was not affected. Although both promoters contained *lacO* sites at the identical position, only an increased level of LacI molecules or three *lacO* sites could sufficiently reduce basal expression in the T5 expression systems. Similar findings were obtained by Lanzer and Bujard [21]. They concluded that the promoter strength was not correlated with effective repression. The host RNAP recognized the A1 promoter only half as efficiently as the T5 promoter [28]. When one *lacO* site was located within the promoter sequence, between the − 10 and − 35 promoter elements, the host RNAP and LacI competed with each other for their respective binding sites, and this competition determined how efficiently promoter activity was controlled by the repressor. The RNAP and T5 promoter form a complex at one of the highest complex-formation rates known in nature [28]. Thus, controlling this promoter requires either a high repressor binding affinity in the operators or a high concentration of repressor molecules.

### Control of recombinant gene expression rate

The control of the transcription rate, also referred to as “tunability”, is used to fine-tune protein production. This fine-tuning is highly relevant in bioprocessing. Optimal bioprocesses are designed to maximally exploit cell synthesizing capacities for long periods to yield correctly folded, processed proteins. Depending on the physical properties and metabolic requirements of the desired product, transcription rates must be adapted to RNA stability, translation efficiency, protein folding, protein transport, and all other interactions in the system.

To evaluate the tunability of the promoter/operator combinations described herein, we tested a series of fed-batch-like microtiter cultivations at varying IPTG levels and benchmarked protein production to the genome-integrated T7 expression system. The range of IPTG concentrations for fully and partially induction with IPTG was determined in a preliminary experiment. The strains B<3lacO-T5> and B3<T7> were induced with following IPTG concentrations: 1.0, 0.5, 0.1, 0.05, 0.01, 0.005 mM IPTG (Additional file 4: Figure S4). Based on these results, we decided on the concentrations 0.005, 0.01 and 0.5 mM IPTG. On-line fluorescence measurements and end-point flow cytometry analyses were used to characterize the different promoter/operator combinations.

**Fig. 3:**
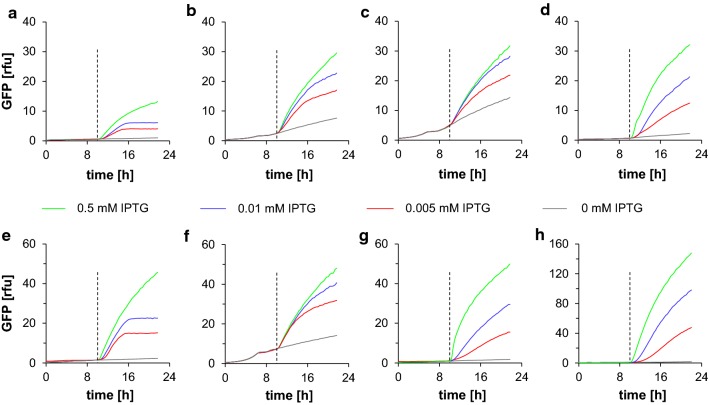
Influence of *lac* operators on expression rate control, shown by the change in on-line GFPmut3.1 fluorescence in fed-batch-like microtiter cultivations. The dashed vertical lines indicate the time of induction. Induction was performed with 0 (gray, not induced), 0.005 (red), 0.01 (blue), or 0.5 mM (green) IPTG. A–D: The T5 promoter is controlled by: **a** three *lacO*, **b** two *lacO*, **c** one *lacO*, and **d** one *lacO*/*lacI*^*Q*^ sequences. **e**–**g** The A1 promoter is controlled by **e** two *lacO*, **f** one *lacO*, and **g** one *lacO*/*lacI*^*Q*^ sequences. **h** The T7 expression system is used as a reference. The Y-axis scale is adjusted to the respective expression rates. The mean relative GFP fluorescence intensity (rfu) represents triplicate samples

Expression systems controlled by one *lacO* site for gene regulation exhibited the highest basal expression and the least pronounced gradation of GFPmut3.1 expression at increasing inducer concentrations (Fig. 3c, f). Although promoters controlled by two *lacO* sites showed sufficiently low basal expression, they also produced less protein at the lower inducer concentrations (Fig. 3b, e). The promoter/operator combinations controlled by 3lacO-T5 and 2lacO-A1 led to a complete stop (plateau) of recombinant GFPmut3.1 production after a certain time, independent of the inducer concentration (Fig. 3a, e). We did not observe this behavior in promoter/operator combinations with only one *lacO* site. The combination of promoters controlled by one *lacO* site and *lacI*^*Q*^ repressor (Fig. 3d, g) and the T7 expression system (Fig. 3h) resulted in the desired system properties, including tunability and low system leakiness.

**Fig. 4:**
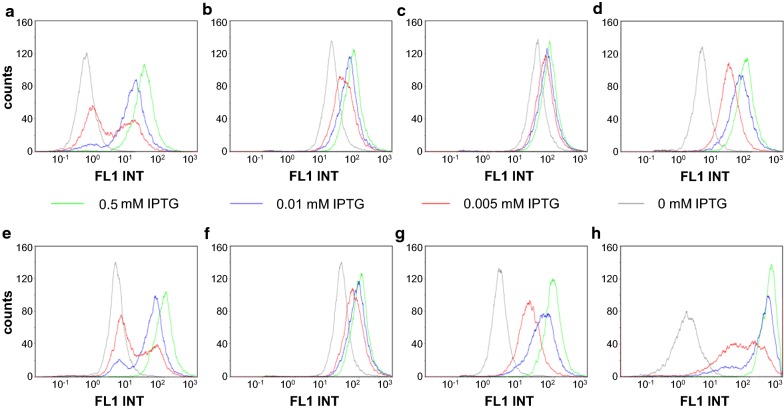
Flow cytometry analysis of single-cell expression of GFPmut3.1. Induction was performed with 0 (gray, not induced), 0.005 (red), 0.01 (blue), or 0.5 mM (green) IPTG. A–D: T5 promoter controlled by: **a** three *lacO*, **b** two *lacO*, **c** one *lacO,* or **d** one *lacO*/*lacI*^*Q*^ sequences. **e**–**g** A1 promoter controlled by: **e** two *lacO*, **f** one *lacO,* or **g** one *lacO*/*lacI*^*Q*^ sequences. **h** The T7 expression system is used as a reference

T7 expression systems exhibit an all-or-none induction phenomenon, where reduced expression in partially induced cultures results from the formation of subpopulations of fully induced and non-induced cells [30]. Therefore, we investigated transcription rate tuning at the cellular level with flow cytometry analyses of all genome-integrated promoter/operator combinations (Fig. 4). We confirmed that the all-or-none phenomenon occurred in genome-integrated T7 expression systems. In fact, we observed a mixture of fully, partially, and non-induced cells, particularly at very low inducer concentrations (Fig. 4h, red line). In the B<2lacO-A1> expression system, flow cytometry analyses revealed that these expression systems stopped GFPmut3.1 production, although the cells continued to grow (Additional file 1: Figure S1, Additional file 2: Figure S2). This result indicated that there were two distinct subpopulations of producing and non-producing cells. We also observed this behavior in B<3lacO-T5> (Fig. 3a, e). But the BQ<1lacO-A1> system showed different behavior. There, the induction of the *gfpmut3.1* gene resulted in a homogenous population at any given IPTG concentration (Fig. 3g). Consequently, this expression system provided proof that the expression rate was controlled on a cellular level.

### Influence of LacI autoregulation on expression rate control

**Fig. 5:**
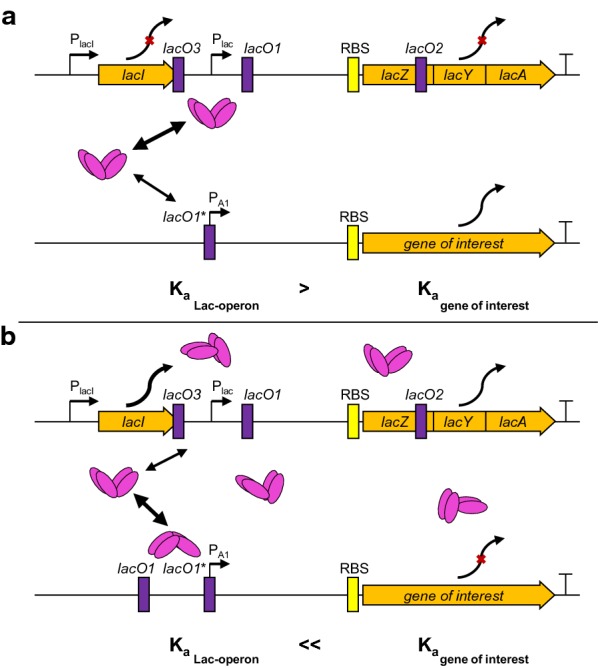
Schematic of *lac* operators in the native *lac* operon (top panels) and its regulation of the gene of interest (bottom panels). Lacl (pink tetramer) production effects are shown, when the promoter for the gene of interest was regulated by **a** one *lac* operator or **b** two *lac* operators, respectively. K_a_ = association constant; red cross = stopped production

We assumed that the complete stop in productivity, observed when the B<3laco-T5> and B<2lacO-A1> systems were partially induced, was associated with the autoregulation of the *lac* repressor. The native *lac* operon is regulated by three *lacO* sites (Fig. 5a). The LacI molecule simultaneously binds to two sites, either *lacO1* and *lacO3* or *lacO1* and *lacO2* [6]. The *lacO3* sequence overlaps with the 3‘end of the *lacI* gene. When LacI binds to *lacO1* and *lacO3*, it causes the DNA to form a loop. This results in truncated *lacI* mRNA molecules, which are degraded by the cell. This autoregulation of LacI production resulted in a constant level of ~ 10 LacI molecules per cell in the absence of an inducer [11, 31, 32].

We hypothesized that, when the binding constant (K_a_) of LacI to the *lacO* sites of the GOI was greater than the binding constant to the *lacO* sites of the *lac* operon, the first LacI molecules, which are not inactivated by IPTG, will preferentially bind to the *lacO* site of the GOI, instead of the *lacO3*/*lacO1* within the *lac* operon. Hence, autoregulation of LacI would not intervene, and LacI molecules would continue to be produced. This would cause the whole system to become overregulated, which would result in a complete stop in production (Fig. 5b).

**Fig. 6:**
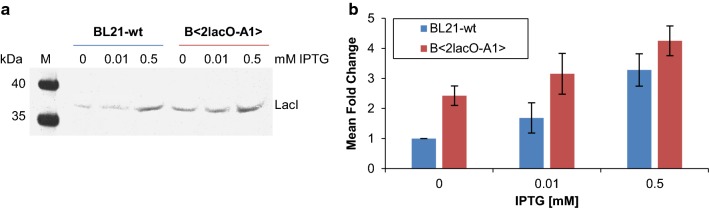
Influence of additional *lacO* sites on cellular LacI concentrations. Proteins of ~ 1.2 × 10^7^ cells were separated with SDS-PAGE and analyzed on western blots, probed with an anti-LacI antibody. **a** Western blot of BL21 wild-type cells and B*<*2lacO-A1*>* cells, which were grown without IPTG, 0.01 mM IPTG, or 0.5 mM IPTG. (M) PageRuler™ Plus Prestained Protein Ladder. **b** Fold changes of band intensities determined in panel **a** are shown relative to the levels observed in 0 mM IPTG BL21-wt cells. Error bars indicate the standard error of the mean (n = 3)

To test this hypothesis, we compared the effect of autoregulation on LacI in B<2lacO-A1> and BL21 wild-type cells (BL21-wt). We estimated the LacI content of non-induced, partially-induced, and fully-induced cells with western blot analyses. The band intensities were quantified and normalized by the cell number (Fig. 6).

In fully induced (0.5 mM IPTG) BL21-wt cells, the number of LacI molecules was 3.3-fold greater than the number observed in non-induced BL21-wt cells. Partial induction with 0.01 mM IPTG only led to a 0.7-fold increase. The 3.3-fold change in fully induced BL21-wt cells was consistent with previous results from Semsey et al. In that study, they measured an average of 15 LacI molecules per cell in the absence of inducer and ~ 40 molecules per cell in fully induced cells [11].

In B<2lacO-A1> cells, LacI numbers in non-induced and partially induced conditions were clearly higher than the numbers observed in uninduced BL21-wt cells. LacI yields were 2.4-fold greater in the absence of inducer and 3.2-fold greater in partially induced cells, relative to uninduced BL21-wt cells. In fully induced cells, LacI yields were 4.3-fold greater than those observed in uninduced BL21-wt cells, which was similar to the yield in fully induced BL21-wt cells.

Although the addition of 0.01 mM IPTG resulted in almost half-maximal GFPmut3.1 expression in B<2lacO-A1> cells (Fig. 3e), it had little or no influence on the LacI levels. This suggested that LacI continued to bind to *lacO1*/*lacO3* in the lac operon; hence, it could maintain autoregulation under these conditions. In the fully induced state, the LacI concentrations are almost the same with a fourfold increase regardless of whether it is the BL21-wt or the B<2lacO-A1> expression system. LacI therefore no longer binds to its operators and thus the expression of LacI itself is no longer inhibited. The small fold change of 4 results from the weak constitutive LacI promoter, which provides about one new mRNA per cell generation [33]. Thus, the high LacI levels in non-induced and partially induced B<2lacO-A1> cells clearly supported our hypothesis that LacI autoregulation impacted the expression rate control in genome-integrated *E. coli* production strains (Fig. 5).

The effect of LacI autoregulation was only observed in genome-integrated, host RNAP-dependent expression systems, which were controlled by two or three *lacO* sites. In contrast, this effect was not observed in plasmid-based, host RNAP-dependent expression systems or in the conventional T7 expression system. This discrepancy might be explained by differences in the balance between *lacO* sites and LacI concentrations. The T7 expression system harbors a second *lacI* gene sequence within its DE3 lysogen, which would, theoretically, double the LacI concentration per cell. The plasmid-based expression systems used in this study were based on the pET plasmid system, which encodes a second *lacI* gene sequence. In turn, depending on the plasmid copy number, that resulted in an extra 15–20 *lacI* gene sequences [34]. However, the effect of LacI autoregulation on partially induced cells was also observed in plasmid-based expression systems, like the *E. coli* pAVEway™ expression system, from Fujifilm Diosynth Biotechnologies (Hillerød, Denmark). In the pAVEway™ expression system, transcription control was enabled by two perfectly symmetric *lac* operators, one positioned upstream and one downstream of the T7A3 promoter. The high affinity of LacI to the symmetric *lacO* sites, combined with the ability to form a DNA loop, resulted in very low basal expression, but also, a complete stop in productivity in partially induced cultures.

Considering the autoregulation of *lac* repressor synthesis, we identified BQ<1lacO-A1> as the σ^70^ promoter/operator combination that fulfilled the desired properties. It showed a high expression rate, negligible basal expression, and true tunability of the expression rate on a cellular level, even at low inducer concentrations, without a complete stop in productivity.

## Conclusion

The regulation of transcription in *E. coli* has recently received considerable attention, because it is the first step in the process of recombinant protein production [35–38]. Transcription control of the GOI allows a cell to divide up its resources between cellular and recombinant proteins in a physiologically balanced manner. Tight and tunable transcription control of the GOI is essential for successful bioprocesses. We showed that, in genome-integrated expression systems, the regulatory elements of the *lac* operon must be well balanced to control σ^70^ promoters. Three *lacO* sites reduced the basal expression, but also reduced the recombinant protein production rate. The perfectly symmetric *lacO* in the ITS hampered the escape of RNAP from the promoter. As shown by Hsu et al. [29], the wild-type ITS of A1 is enriched in purines, and it displayed one of the best promoter escape efficiencies. Promoters that contained only one *lacO* site exhibited a considerably higher promoter strength, but also higher leakiness. In promoter/operator combinations that contained two *lacO* sites, when the two *lacO1* sites were located within a distance of 62 bp, they exhibited very strong binding affinity with the repressor molecule, which prevented LacI autoregulation. These conditions resulted in a complete stop in productivity in partially induced cells. However, we did not consider that all promoters with two *lacO* sites were unsuitable, in general. The binding affinity can be reduced by using less symmetric *lacO* sites, like *lacO3* or *lacO2*, or by varying the distance between the *lacO* sites [8, 23]. The combination of one *lacO1* site and the *lacI*^*Q*^ promoter (which increased LacI levels) resulted in high GFPmut3.1 expression rates, low basal expression, and true tunability on a cellular level. Thus, we concluded that this novel genome-integrated, host RNAP-dependent expression system would be advantageous for the production of challenging proteins, because it obviates the plasmid-mediated metabolic load, and it confers true tunability on a cellular level.

## Methods

### Strains and culture conditions

*Escherichia coli* K-12 NEB5-α [*fhuA2Δ(argF*-*lacZ)U169 phoA gln V44 Φ80 Δ(lacZ)M15 gyrA96 recA1 relA1 endA1 thi*-*1 hsdR17*] (New England Biolabs [NEB], Ipswitch, MA, USA) was used for all cloning procedures. Linear DNA cartridges were integrated into the bacterial chromosome at the attTN7 site of *E. coli* BL21 [*fhuA2 [lon] ompT gal [dcm] ΔhsdS*] (NEB). For reference experiments, the same strains were transformed with the respective plasmids, except that they carried the sequence for the soluble protein, GFPmut3.1, which was used as a recombinant model protein [25].

The strains were cultivated in the BioLector micro-fermentation system, in 48-well Flowerplates^®^ (m2p-labs, Baesweiler, Germany), as described by Török et al. [39]. We used a synthetic Feed in Time (FIT), fed-batch medium, with 1 g/L glucose and 16.5 g/L dextran as carbon sources (m2p-labs GmbH, Baesweiler, Germany). Additionally, the medium contained (g/L): 27.40 MOPS, 6.54 (NH_4_)_2_SO_4_, 1.96 K_2_HPO_4_, 1.96 trisodium citrate·2H_2_O, 1.31 Na_2_SO_4_, 0.65 NH_4_Cl, 0.33 MgSO_4_·7H_2_O, and 0.0065 Thiamin·HCl.

The trace element solution contained (mg/L): 0.36 ZnSO_4_·7H_2_O, 0.33 CuSO_4_·5H_2_0, 0.20 MnSO_4_·H_2_O, 27.30 FeCl_3_·6H_2_O, 21.84 Titriplex III, 0.36 CoCl_2_·6H_2_O, and 1.31 CaCl_2_·2H_2_O. Immediately prior to inoculation, 0.6% (v/v) glucose releasing enzyme mix (EnzMix) was added. Expression levels were monitored at an excitation wavelength of 488 nm and an emission wavelength of 520 nm. The signals are expressed in relative fluorescence units [rfu]. The cycle time for all parameters was 20 min. The initial cell density was equivalent to 0.3 optical density at 600 nm (OD_600_). For inoculation, a deep-frozen (− 80 °C) working cell bank (OD_600_ = 3.5) was thawed, and the biomass was harvested by centrifugation (7500 rpm, 5 min). Cells were washed with 500 μL of the corresponding medium to remove residual glycerol. Next, cells were centrifuged, and the pellets were resuspended in the total cultivation medium. All cultivations were prepared in three replicates at 30 °C for 22 h. Recombinant gene expression was induced with 0.005 mM, 0.01 mM, or 0.5 mM IPTG at 10 h after the start of cultivation.

### Construction and characterization of promoter/operator combinations

Basic cloning methods, like restriction endonuclease digestions, agarose gel electrophoresis, plasmid engineering, and transformation of *E. coli* plasmids, were carried out according to Sambrook et al. [40]. For the integration of the *lacI*^*Q*^ promoter into *E. coli* BL21 (NEB), we constructed the plasmid, pETAmp-lacIq. This plasmid contained the ampicillin resistance gene (Amp), flanked by FRT sites [41], and the *lacI* gene controlled by the *lacI*^*Q*^ promoter [33]. The pBR322 ori and the *lacI* gene were amplified from pET30a with the overhang PCR technique to add a C → T mutation within the *lacI* promoter. The linear *lacI*^*Q*^ DNA cartridge for genome-integration was amplified with the Q5^®^ High-Fidelity DNA Polymerase (NEB), according to the manufacturer’s instructions. Integration into the bacterial chromosome occurred at the *lac* operon site of *E. coli* BL21, which carries the pSIM5 plasmid, as described by Sharan et al. [42]. This strain was designated BL21^Q^. The sequences of the T7_A1_ and the T5_N25_ promoters were adopted from Lanzer and Bujard [21] (designated as P_A1/04_ and P_N25/04_, respectively). These promoters contained a 2-bp truncated *lacO1* sequence, inserted between the − 10 and − 35 region, upstream of the promoter. These promoters were purchased as gBlocks^®^ Gene Fragments (Integrated DNA Technologies, IA/USA), which contained a 5′ spacer sequence from pET30a and the restriction sites, SphI (5′) and XbaI (3′); these were subsequently cloned into the pET30a-cer-tZENIT-GFPmut3.1 backbone. The tZENIT terminator was described elsewhere [27]. A second *lacO1* sequence, 62 bp upstream of the first *lacO1* sequence, was added via the overhang PCR technique. The 3lacO-T5 promoter/operator combination was adopted from the pJexpress 401–406 (T5) vector from ATUM (Newark, NJ, USA). Linear DNA cartridges were integrated into the bacterial chromosome at the attTN7 site of *E. coli* BL21 or *E. coli* BL21^Q^.

### GFPmut3.1 off-line expression analysis and quantification

In addition to on-line measurements of recombinant GFPmut3.1, expressed in rfus, we performed absolute quantifications with ELISA, according to Reischer et al. [43]. Inclusion body formation was analyzed with SDS-PAGE, as previously described [44] and fractions of soluble and insoluble protein were estimated with ImageQuant TL software (GE Healthcare, Chicago, IL, USA).

### Flow cytometry

A Gallios flow cytometer (Beckman Coulter, Brea, CA, USA) was used to determine the fraction of GFPmut3.1-producing cells. Cells were harvested 12 h after induction, then diluted 1:2025 in PBS. GFPmut3.1 fluorescence was excited with an OPSL Sapphire Laser at 488 nm, and the subsequent emission was measured with the FL1 Channel (505–545). Data were recorded for 15,000 cells per sample at ~ 300 events/sec. Analyses were performed with Kaluza analysis software (Beckman Coulter).

### Analysis of LacI with western blots

Cell extracts were prepared with ~ 1.2 × 10^7^ BL21-wt and B<2lacO-A1> cells, respectively, and proteins were separated with SDS-PAGE, as previously described. After separation, the proteins were blotted with the iBlot^®^ Dry Blotting System, according to the manufacture’s instructions (Invitrogen™/Thermo Fisher Scientific, Waltham, MA, USA). Subsequently, proteins were blocked for 4 h at room temperature with 3% nonfat dry milk in PBST (1x PBS Dulbecco and 0.05% Tween 20). The blots were then incubated with primary antibody (1:1000 anti-LacI Antibody, clone 9A5; Sigma-Aldrich/Merck, St. Louis, MO, USA) for 1 h at room temperature. Blots were then incubated with alkaline phosphatase-conjugated secondary antibody (1:2000 Anti-Mouse IgG, whole molecule, Sigma A5153; Sigma-Aldrich) for 1 h at room temperature. Blots were developed with SigmaFAST™ BCIP^®^/NPT tablets (Sigma-Aldrich) according to the manufacturer’s instructions. Band intensities were quantified with ImageQuant TL software (GE Healthcare, Chicago, IL, USA).

## Supplementary information

**Additional file 1: Figure S1.** Growth characteristics of genome-integrated expression systems with different promoter/operator combinations. Cells were grown in enzymatic glucose release media in micro-titer fermentations over a period of 22 h. The dashed vertical lines indicate the time of induction with 0.5 mM IPTG. (A, B) Biomass trends (CDM) and (C, D) growth rates (µ) are shown for (A, C) induced and (B, D) non-induced cells. The mean values of triplicates are shown. The promoter/operators are defined in Fig. 1.

**Additional file 2: Figure S2.** Growth characteristics of plasmid-based expression systems with different promoter/operator combinations. Cells were grown in enzymatic glucose release media in micro-titer fermentations over a period of 22 h. The dashed vertical lines indicate the time of induction with 0.5 mM IPTG. (A, B) Biomass trends (CDM) and (C, D) growth rates (µ) are shown for (A, C) induced and (B, D) non-induced cells. The mean values of triplicates are shown. The promoter/operators are defined in Fig. 1.

**Additional file 3: Figure S3.** Solubility analysis of GFPmut3.1. SDS-PAGE images show soluble (S) and insoluble (I) fractions of proteins produced under the indicated *lacO*-promoter combinations in genome-integrated (indicated with pointed brackets <>) and plasmid-based (indicated with round brackets ()) expression systems.

**Additional file 4: Figure S4.** Determination of IPTG concentrations for full and partial induction. The dashed vertical lines indicate the time of induction. Induction was performed with 0 (gray, not induced), 0.005 (red), 0.01 (blue), 0.05 (orange), 0.1 (violet), 0.5 (green) or 1.0 (black) mM IPTG. (A) B<3lacO-T5>. (B) B3<T7>. The mean relative GFP fluorescence intensity (rfu) represents triplicate samples.

## Data Availability

All data generated or analyzed during this study are included in this published article and its Additional files.
