# Supplementary figures and images for: Escherichia coli σ70 promoters allow expression rate control at the cellular level in genome-integrated expression systems
Source: Microb Cell Fact. 2020 Mar 5;19:58. doi: 10.1186/s12934-020-01311-6 (PMC7059391; doi:10.1186/s12934-020-01311-6)

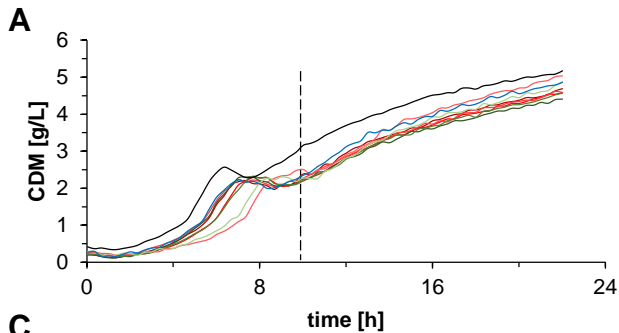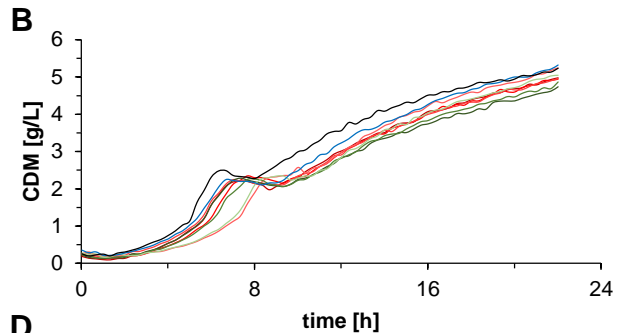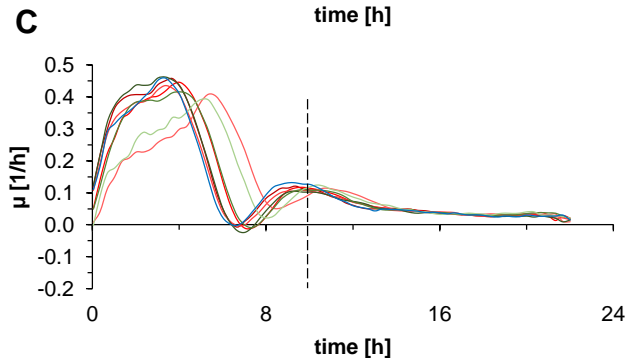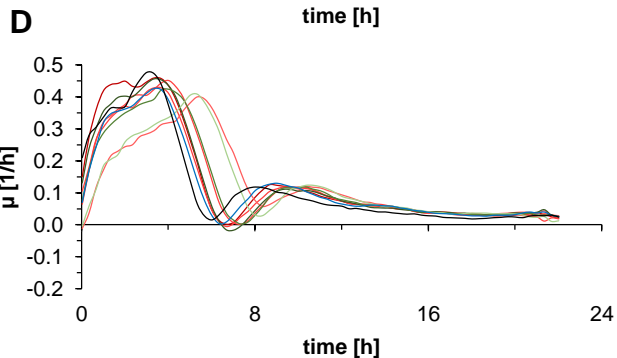

— B<3lacOT5-GFP>  
— BQ<1lacOT5-GFP>  
— BQ<1lacOA1-GFP>

— B<2lacOT5-GFP>  
— B<2lacOA1-GFP>  
— B3<T7-GFP>

— B<1lacOT5-GFP>  
— B<1lacOA1-GFP>  
— BQ-wt

Supplement: Supplementary file 1 — Additional file 1: Figure S1. Growth characteristics of genome-integrated expression systems with different promoter/operator combinations. Cells were grown in enzymatic glucose release media in micro-titer fermentations over a period of 22 h. The dashed vertical lines indicate the time of induction with 0.5 mM IPTG. (A, B) Biomass trends (CDM) and (C, D) growth rates (µ) are shown for (A, C) induced and (B, D) non-induced cells. The mean values of triplicates are shown. The promoter/operators are defined in Fig. 1. [file 12934_2020_1311_MOESM1_ESM.pdf]

**A**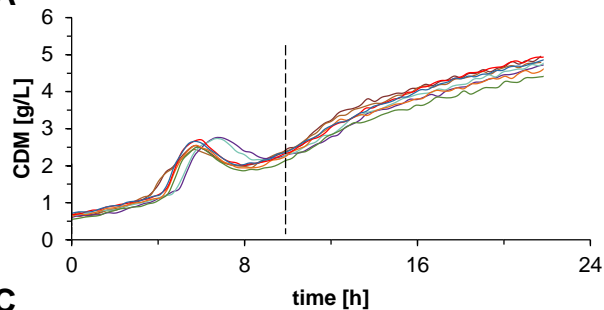**B**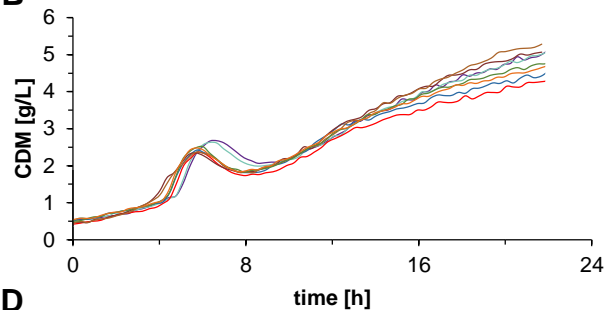**C**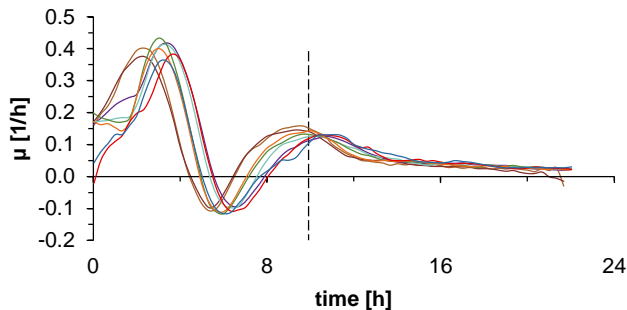**D**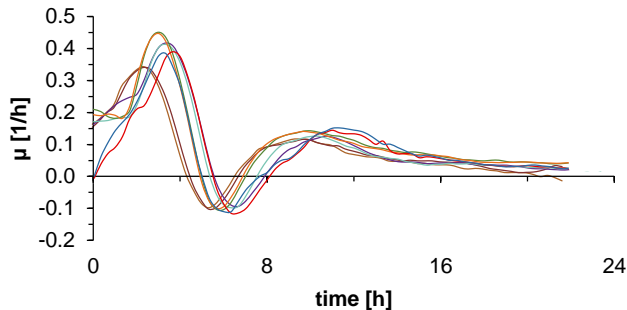

— B(3lacOT5-GFP)  
— BQ(1lacOT5-GFP)  
— BQ(1lacOA1-GFP)

— B(2lacOT5-GFP)  
— B(2lacOA1-GFP)  
— B3(T7-GFP)

— B(1lacOT5-GFP)  
— B(1lacOA1-GFP)

Supplement: Supplementary file 2 — Additional file 2: Figure S2. Growth characteristics of plasmid-based expression systems with different promoter/operator combinations. Cells were grown in enzymatic glucose release media in micro-titer fermentations over a period of 22 h. The dashed vertical lines indicate the time of induction with 0.5 mM IPTG. (A, B) Biomass trends (CDM) and (C, D) growth rates (µ) are shown for (A, C) induced and (B, D) non-induced cells. The mean values of triplicates are shown. The promoter/operators are defined in Fig. 1. [file 12934_2020_1311_MOESM2_ESM.pdf]

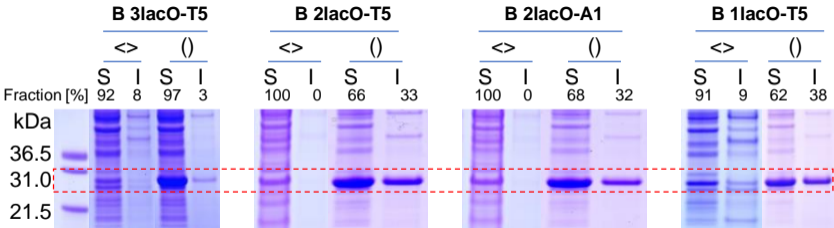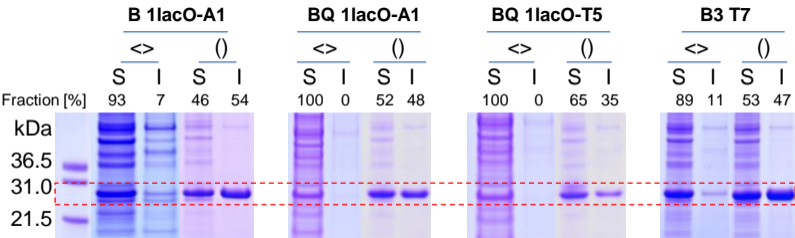

Supplement: Supplementary file 3 — Additional file 3: Figure S3. Solubility analysis of GFPmut3.1. SDS-PAGE images show soluble (S) and insoluble (I) fractions of proteins produced under the indicated lacO-promoter combinations in genome-integrated (indicated with pointed brackets <>) and plasmid-based (indicated with round brackets ()) expression systems. [file 12934_2020_1311_MOESM3_ESM.pdf]

**A**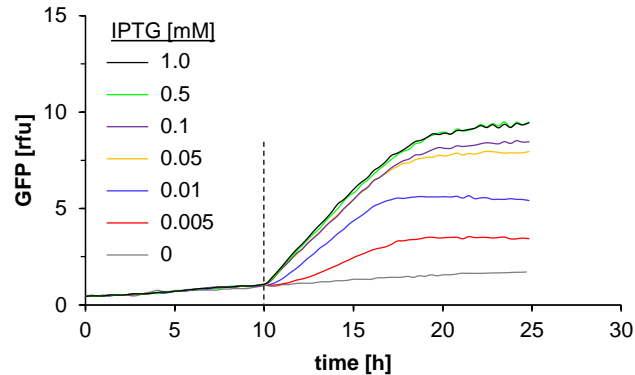**B**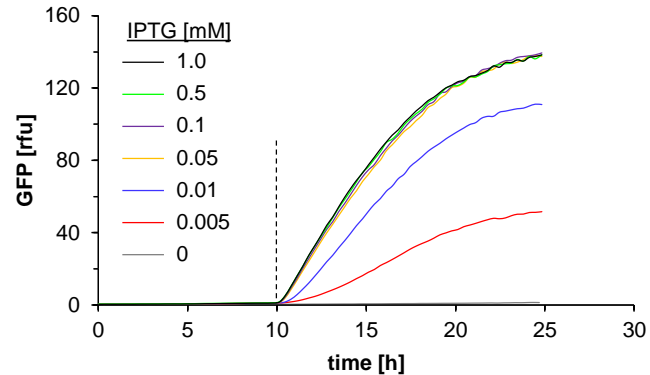

Supplement: Supplementary file 4 — Additional file 4: Figure S4. Determination of IPTG concentrations for full and partial induction. The dashed vertical lines indicate the time of induction. Induction was performed with 0 (gray, not induced), 0.005 (red), 0.01 (blue), 0.05 (orange), 0.1 (violet), 0.5 (green) or 1.0 (black) mM IPTG. (A) B<3lacO-T5>. (B) B3. The mean relative GFP fluorescence intensity (rfu) represents triplicate samples. [file 12934_2020_1311_MOESM4_ESM.pdf]
